# A novel emulsion PCR method coupled with fluorescence spectrophotometry for simultaneous qualitative, quantitative and high-throughput detection of multiple DNA targets

**DOI:** 10.1038/s41598-018-36981-1

**Published:** 2019-01-17

**Authors:** Yanan Du, Xiao Zhao, Binan Zhao, Yan Xu, Wei Shi, Fangfang Ren, Yangyang Wu, Ruili Hu, Xiaorui Fan, Qi Zhang, Xiaoxia Zhang, Wanjing Zhang, Wenjing Wu, Bin Shi, Huanzhen Zhao, Kai Zhao

**Affiliations:** 1 Biotechnology Research Institute, Shanghai Academy of Agricultural Sciences, 2901 Beidi Road, Shanghai, 201106 China; 2 Key Laboratory of Agricultural Genetics and Breeding, Shanghai Academy of Agricultural Sciences, 2901 Beidi Road, Shanghai, 201106 China; 3 College of Life and Environment Sciences, Shanghai Normal University, 100 Guilin Road, 200234 Shanghai, China; 4 Department of Clinical Medicine, XuZhou Medical University, 209 Tongshan Road, 221004 Xuzhou, China; 5 Shanghai Bio-full Biotech Co.,Ltd, 2901 Beidi Road, Shanghai, 201106 China

## Abstract

We constructed and validated a novel emulsion PCR method combined with fluorescence spectrophotometry (EPFS) for simultaneous qualitative, quantitative and high-throughput detection of multiple DNA targets. In a single reaction set, each pair of primers was labeled with a specific fluorophore. Through emulsion PCR, a target DNA was amplified in droplets that functioned as micro-reactors. After product purification, different fluorescent-labeled DNA products were qualitatively analyzed by the fluorescent intensity determination. The sensitivity and specificity of the system was examined using four kinds of genetically modified (GM) maize. The qualitative results revealed high specificity and sensitivity of 0.5% (w/w). In addition, the quantitative results revealed that the absolute limit of detection was 10^3^ copies, showing good repeatability. Moreover, the reproducibility assays were further performed using four foodborne pathogenic bacteria to further evaluate the applicability of the system. Consequently, the same qualitative, quantitative and high-throughput results were confirmed with the four GM maize. To sum up, the new EPFS system is the first analytical technology of this kind that enables simultaneous qualitative, quantitative and high-throughput analysis of multiple genes.

## Introduction

The increases in the number of biomolecular samples that need to be analyzed generate a great demand for a high-throughput detection method. Traditional nucleic acid based technologies can usually detect only one target gene in one reaction. When dealing with complex nucleic acid samples, multiple reactions need to be performed separately, which is costly and time consuming. Therefore, it is of great importance to develop an economical and more effective technique that would enable simultaneous qualitative and quantitative detection of these samples. Indeed, several multiplex detection methods of target genes have been developed and employed in the fields of food/feed identifications^1–4^, medical diagnostics^5–7^, and large scale sequencing^8–10^.

Although conventional multiplex PCRs have always been used for amplification of nucleic acid samples, problems such as preferential amplification of shorter DNA templates, interference of multiple primer pairs and limited substrates limit their wide use in quantitative and high-throughput research^11,12^. Real-time quantitative PCRs have been widely used to quantify target genes with high sensitivity, specificity and a wide dynamic range^13–16^. Nonetheless, their throughput is low because of the limited number of channels in the real-time system. DNA microarray is a high-throughput approach used for analyzing complex nucleic samples that have limited feasible availability due to the complicated procedure and expensive consumption. Poor linearity is another weakness of DNA microarray that limits its use in quantitative analysis^17^. Therefore, developing a novel qualitative, quantitative and high-throughput method for detecting multiple biological genes is of great importance.

The multiplex emulsion PCR has been developed for high-throughput simultaneous amplification of several DNA targets, either used alone^18,19^ or combined with other methods^4,9^. In emulsion PCR, the different DNA targets are compartmentalized in millions of micro-reservoirs and parallelly amplified in a water-in-oil (w/o) emulsion. Emulsion PCR, alleviates nonspecific amplification in conventional multiplex PCR, increases the throughput and reduces the regent and sample consumption.

In this paper, we constructed and validated a novel high-throughput detection method based on combining emulsion PCR and fluorescence spectrophotometry (EPFS) for qualitative, quantitative and high-throughput detection of multiple biological genes. The new method uses the primer pairs labeled with different fluorophores, so that the multitarget DNAs in one mixed sample could be amplified in a single reaction set; and then qualitatively and quantitatively analyzed on Infinite M1000 PRO. Four genetically modified (GM) maize lines and four foodborne pathogenic bacteria were qualitatively and quantitatively tested obtaining satisfactory results. The EPFS method we developed provides a new approach for qualitative, quantitative and high-throughput analysis of multitarget DNAs for a broad range of biological samples.

## Materials and Methods

### Materials

GM maize flour i.e. Bt176, GA21, NK603 and TC1507, were supplied by Monsanto Company (St. Louis, MO). Non-transgenic seeds (maize, soybean, rapeseed, wheat) were purchased from a local market in Shanghai, China. The genomic DNAs of four common foodborne pathogenic bacteria i.e. Salmonella, Listeria monocytogenes (L. monocytogenes), Escherichia coli (E. coli) and Staphylococcus aureus (S. aureus), and the corresponding plasmids used for reproducibility assay were obtained from our laboratory.

### Sample Preparation

The genomic DNAs of all plant materials were extracted and purified using a mini-plant genomic DNA extraction kit (Shanghai Bio-ful Biotech Co., Ltd., Shanghai, China). The DNA concentration and quality were estimated using a NanoDrop 1000 UV-Vis spectrophotometer (NanoDrop Technologies, LLC, Wilmington, DE). To test the 4-plex sensitivity, a series of DNA solution were obtained by mixing the GM maize flour with non-GM maize flour. The final relative content was 10%, 5%, 1% and 0.5% (w/w) for each of the four GM maizes, respectively. Event-specific genes of the four GM maize were used for the construction of standard plasmids as calibrators; plasmids were serially diluted from 10^7^ to 10^3^ and amplified for quantitative detection. Easy dilution (TaKaRa biotechnology Co., Ltd, Dalian, China) was used to dilute the standard plasmids to avoid DNA loss due to adsorption to the tube walls.

### Set-up of EPFS Reaction

#### The Selection Principle of Fluorophores and Primer Labeling

According to the experimental data and rigorous calculation supplied by ThermoFisher Scientific (ThermoFisher Scientific Co., Ltd., Shanghai, China), when the stoke shift of a fluorophore was higher than the sum of excitation and emission bandwidth of the microplate reader, the optimal excitation and emission wavelength were set to get stronger signal and to prevent overlap. Based on the parameter characteristics of the multi-mode microplate reader Varioskan Flash reader (Thermo Scientific), the excitation bandwidth was 5 nm and the emission bandwidth was 12 nm. Consequently, the fluorophores with stokes shift that was >17 nm, were more easy to detect. Furthermore, the excitation spectrum and the emission spectrum of two adjacent fluorophores should not overlap.

Based on the above listed principles, twelve fluorophores were selected among 300 fluorophores. Almost 20 fluorophores can be tested simultaneously without affecting the fluorescence strength and sensitivity when the excitation and emission wavelength are properly adjusted. In this study, four fluorophores were elaborately selected. In addition, four pairs of GM maize primers and foodborne pathogenic bacteria primers were labeled with the selected fluorophores and synthesized (ThermoFisher Scientific Co., Ltd., Shanghai, China) to amplify the targeted DNA. A pair of primers of internal reference gene (IVR) of maize were also labeled with another fluorophore for detection of the simulated samples. The primer sequences and the labeled fluorophores are shown in Table 1.

**Table 1:** Primer sequences and the labeled fluorophores.

Primer name	Sequences	Fluorophores	Excitation Wavelength	Emission Wavelength
Bt176-F	5′-AAGCACGGTCAACTTCCGTAC-3′	5′ FAM	470 nm	522 nm
Bt176-R	5′-TCGACTTTATAGGAAGGGAGAGG-3′			
GA21-F	5′-CTTATCGTTATGCTATTTGCAACTTTAGA-3′	5′HEX	505 nm	556 nm
GA21-R	5′-TGGCTCGCGATCCTCCT-3′			
NK603-F	5′-CGGTACCAAGCTTTTATAATAGTAG-3′	5′ ROX	550 nm	602 nm
NK603-R	5′-CTAGTCTGTTATGGTTCGAG-3′			
TC1507-F	5′-GCCAGTTAGGCCAGTTACCCA-3′	5′ NED	530 nm	575 nm
TC1507-R	5′-CAAGATCAAGCGGAGTGAGG-3′			
IVR-F	5′-GTATCACAAGGGCTGGTACC-3′	5′ CY5	630 nm	665 nm
IVR-R	5′-CCGTGTAGAGCATGACGATC-3′			
Salmonella-F	5′-TTGTGCCGAAGAGCCGGCGT-3′	5′ FAM	470 nm	522 nm
Salmonella-R	5′-TTGCGAATAACATCCTCAAC-3′			
L. monocytogenes-F	5′-GGTTTAGCTTGGGAATGGTG-3′	5′HEX	505 nm	556 nm
L. monocytogenes-R	5′-GATAAAGCGTAGTGCCCCAG-3′			
E. coli-F	5′-CGTCGTGTCTGCTAAAAC-3′	5′ ROX	550 nm	602 nm
E. coli-R	5′-GGTTGCTTGCGTTTGAGAC-3′			
S. aureus-F	5′-AAATCCAGCACAACAGGAAACGACACA-3′	5′ NED	530 nm	575 nm
S. aureus-R	5′-ATCTCCGGCCATAATTGGTGGCACT-3′			

#### The Composition of Emulsion

The aqueous phase for the singleplex GM maize tests consisted of 1 × PCR buffer, 10 g/L BSA, 0.2 mM dNTPs, 0.4 μM of forward and reverse primers, 26 U Taq DNA polymerase and 10.4 μL template DNA in a total volume of 260 μL. Singleplex emulsion PCR assays for testing of the GM maize were performed to evaluate the specificity of the bipartite primers using the following program: 95 °C for 5 min; 35 cycles at 94 °C for 30 s, 57 °C for 40 s, 68 °C for 35 s; a final extension at 68 °C for 7 min.

For simultaneous detection of four GM maize, multiplex emulsion PCR pooled four singleplex emulsion PCR into one reaction. The aqueous phase contained the following reagents: 1 × PCR buffer, 10 g/L BSA, 0.2 mM dNTPs, 0.2 μM for each pair of forward and reverse primers, 104 U for Taq polymerase and 10.4 μL template DNA in a total volume of 260 μL. The reaction protocols were as follows: 94 °C for 5 min; 35 cycles at 94 °C for 30 s, 57 °C for 30 s, 72 °C for 30 s; a final extension at 72 °C for 7 min.

Multiplex PCR aqueous phase for four foodborne pathogenic bacteria consisted of 10 × PCR buffer, 10 g/L BSA, 0.2 mM Mg^2+^, 0.3 mM dNTPs, 0.2 μM for each pair of forward and reverse primers, 16.25 U Taq DNA polymerase and 6.5 μL template DNA in a total volume of 260 μL. The multiplex emulsion PCR protocols for foodborne pathogenic bacteria were as the follows: 94 °C for 5 min; 35 cycles at 94 °C for 30 s, 60 °C for 30 s, 72 °C for 30 s; a final extension at 72 °C for 7 min.

The oil-surfactant mixture of emulsion PCR was prepared by mixing the 4.5% span %, 0.4% tween 80, 0.05% triton X-100 and 95.05% mineral oil at 1000 r.p.m for 2 h.

#### Emulsion PCR Amplification

The emulsion PCR was carried out according to previously described method^19^ with some improvements. At 1000 r.p.m, the water-in-oil emulsions were obtained by adding 200 ul of the aqueous phase to 400 ul of the oil phase drop-wise (time interval is 6 s). Meanwhile, a magnetic stirring bar (3 × 8 mm)was stirred in a disposable 2 ml ampoule at 25 °C. Over the period of 5 min, the water-in-oil mixture was processed into discrete encapsulation as micro-reservoir for individual reaction in parallel. Then, the obtained w/o emulsion was delivered into PCR tubes as 12 aliquots of 50 μL for further thermal cycling and the residual 50 ul of the aqueous phase were used as a no-emulsified control. The emulsion PCRs were performed using the corresponding protocols described above.

Prior to the standard analyzing protocol for the PCR products, the resulting emulsion mixtures were pooled and centrifuged at 16200 g for 5 min. Approximately 140 ul of lower aqueous phase contained fluorescent-labeled amplicons were then obtained and purified to remove the residual labeled primers and other disruptive substances. An AxyPrep PCR Cleanup Kit (Axygen Scientific, Inc.) was used for purification in accordance with the manufacturer’s instructions except for elution with 100 μL elution buffer which was performed to get the final amplicons for subsequent analysis.

### Infinite 200^®^ PRO and Magellan analysis

The multiple fluorescent-labeled amplicons were detected and analyzed using Infinite M1000 PRO (Tecan Austria GmbH, Grödig, Austria) with narrow bandwidths and the matching Magellan standard software according to the manufacturer’s instructions. First, the purified amplicons from emulsion PCR were directly transferred to a disposal, black, flat-bottom, 384-well Fluorotrac^TM^ 200 plates (Greiner Bio-One Suns Co., Ltd, Beijing, China). Fluorescent intensity scan were performed to determine the optimal excitation and emission wavelength (Table 1). Due to the intrinsic excitation and emission wavelength of fluorophores, four fluorescent-labeled amplicons could be detected simultaneously using the determination of fluorescence intensity to obtain the relative fluorescence units (RFU).

#### Calculation of Threshold

Following the EPFS method we developed, blank (no template control, NTC) samples were detected 1000 times to determine a threshold necessary to distinguish between negative and positive results. Based on statistical analysis of the blank RFU, data distribution was normal.

As a threshold signal value, a level mapping to $\bar{{\rm{x}}}$ + 4 SD of blank was chosen as a criterion for a positive signal. The probability density of the normal distribution is in equation (1):

1 $$f(x)=\frac{1}{\sqrt{2{\rm{\pi }}}{\rm{\sigma }}}\exp (-\frac{{(x-{\rm{\mu }})}^{2}}{2{{\rm{\sigma }}}^{{\rm{2}}}})$$

where μ is the mean or expectation of the distribution and also its median and mode ($\bar{{\rm{x}}}$), σ is the standard deviation (SD), σ^2^ is the variance, and x is the independent variable.

#### Qualitative, Quantitative and High-Throughput Detection

For testing the specificity of singleplex EPFS assay, fluorescence intensity was performed on the resulting FAM labeled amplicons (BT176’s case) using the following parameters: excitation wavelength λ = 470 nm and emission wavelength λ = 522 nm. The manual gain was set to 100. All the generated data was saved in Excel for further analysis. The amplicons RFU greater than the calculated threshold were considered positive, which meant that the determined samples contained GM maize (BT176). The sensitivity of singleplex EPFS assay were evaluated on a series of GM maize with different relative content (w/w). The lowest positive level was the relative limit of detection (LOD). A series of standard samples (serially diluted plasmids) were detected to construct the standard curve for quantitative analysis of the unknown samples. A gradient of standard samples and concentration was set and the linear regression analysis, the correlation coefficient and blank reduction were adopted to provide a net RFU increase which were supposed to be proportional to DNA copies in the resulting products. Multiplex EPFS assays were achieved by simultaneously performing four determination of fluorescence intensity for qualitative and quantitative analysis of four GM maize with high throughput.

## Results

### The Principle of EPFS

**Figure 1:**
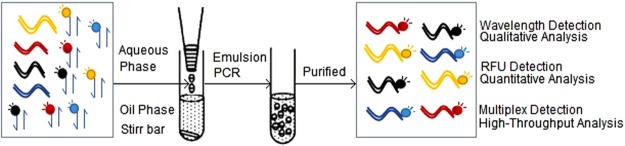
Schematic of EPFS method. Primer pairs are labeled with specific fluorophores in the same reaction set, and template fragments are amplified and labeled with the same fluorophore in the minute aqueous droplets through the emulsion PCR. The purified amplicons tagged with different fluorophores was detected for qualitative, quantitative and high-throughput analysis.

In EPFS method, each of the fluorophores has its own intrinsic excitation and emission wavelength that can be identified and measured by Infinite M1000 PRO. Each pair of primers specific to a target sequence was labeled with a specific fluorophore that did not interfere with other fluorophores in the same reaction set (Fig. 1). Through the emulsion PCR, a target DNA was amplified and labeled with the same fluorophore. The products were then purified by PCR cleanup kit to remove the redundant primers and other disruptive substances. Finally, the resulting amplicons tagged with different fluorophores were qualitatively analyzed by the fluorescent intensity determination. A series of standard samples were also amplified and the RFU was measured to construct standard curves. Using this new system, we were able to simultaneously amplify different target samples in a single PCR tube.

### BT176, GA21, NK603 and TC1507 Singleplex Assay

#### Specificity Assay and Qualitative Detection

**Figure 2:**
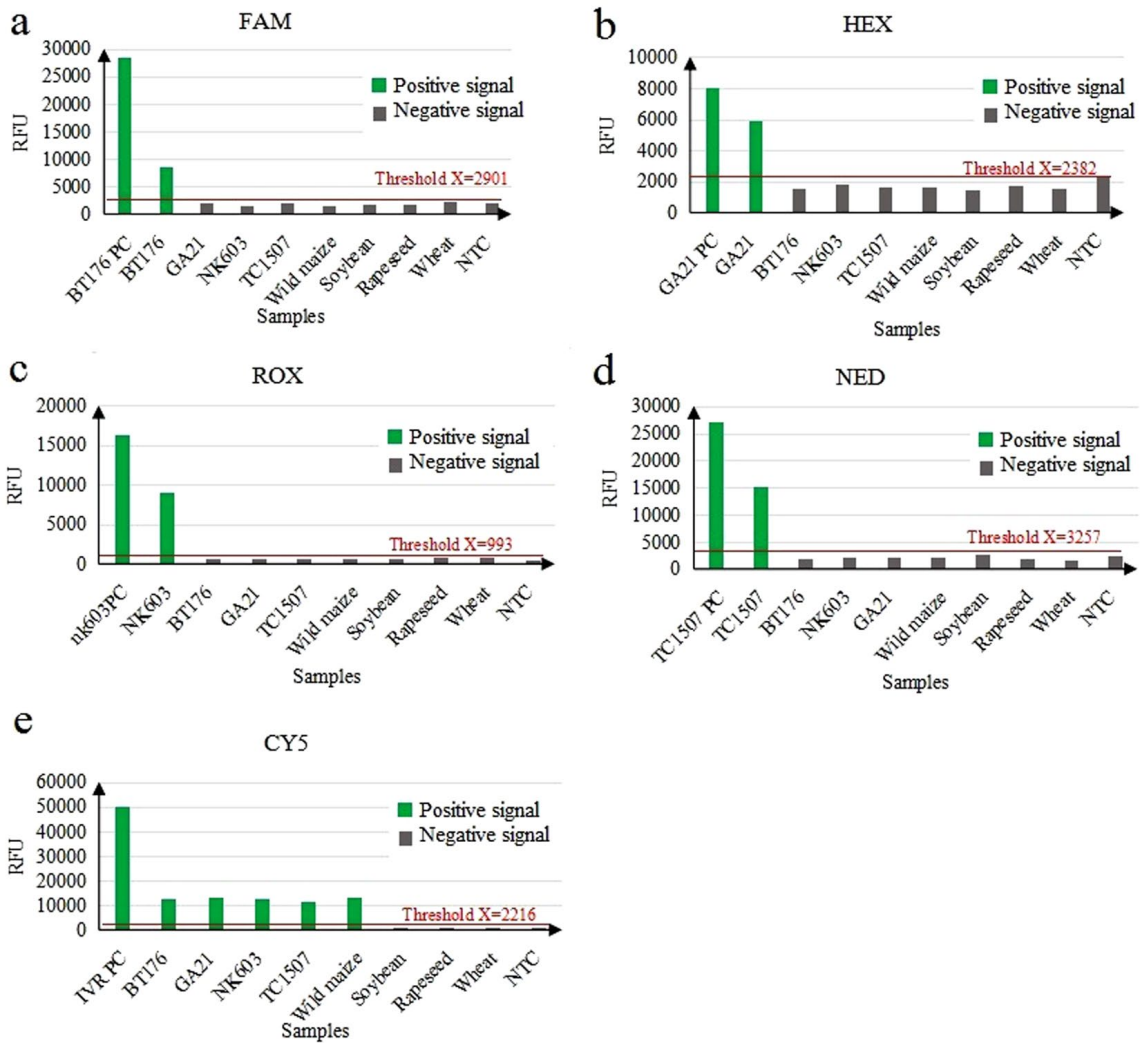
Specificity of the singleplex EPFS assay. (**a**) The specificity assay for detection of BT176 GM maize. The forward primer of BT176 were labeled with FAM to amplify different DNA samples, and FAM-labeled amplicons were analyzed. (**b**) The specificity assay for detection of GA21 GM maize. The forward primer of GA21 were labeled with HEX to amplify different DNA samples and HEX-labeled amplicons were analyzed. (**c**) The specificity assay for detection of NK603 GM maize. The forward primer of NK603 were labeled with ROX to amplify different DNA samples and ROX-labeled amplicons were analyzed. (**d**) The specificity assay for detection of TC1507 GM maize. The forward primer of TC1507 were labeled with NED to amplify different DNA samples and NED-labeled amplicons were analyzed. (**e**) The specificity assay for detection of IVR gene of maize. The forward primer of IVR gene were labeled with CY5 to amplify different DNA samples and CY5-labeled amplicons were analyzed. RFU: relative fluorescence units; PC: positive control; NTC: no template control.

The specificity of the singleplex EPFS method was carefully evaluated by qualitative PCR on genomic DNAs in eight samples including BT176 maize, GA21 maize, NK603 maize, TC1507 maize, non-GM maize, soybean, rapeseed, wheat, positive control (plasmids) and no template control (NTC,ddH_2_O). As shown in Fig. 2a, the positive signals were detected when using forward and reverse primers to amplify BT176 genomic DNA and the BT176 positive control (BT176 plasmid); while, no amplification signal was detected in other samples, including NTC. In addition, our data indicated that the primer pairs of GA21 (Fig. 2b), NK603 (Fig. 2c), TC1507 (Fig. 2d), and maize IVR (Fig. 2e) could only amplify the expected target DNA sequences without generating nonspecific amplification. To sum up, the above data suggested that singleplex EPFS assay has a high specificity for detecting GM maize and maize IVR gene.

#### Sensitivity Assay

A series of DNA solution with different relative GMO content (fortified at 10%, 5%, 1%, 0.5% and 0.1% (w/w)) were used as templates to further investigate the sensitivity of the system (Fig. S1 for singleplex sensitivity). We were able to detect BT176 (Fig. S1a) down to 0.5% (w/w) GM content. The relative LOD of BT176, GA21 maize (Fig. S1b), NK603 (Fig. S1c) and TC1507 (Fig. S1d) were 0.5%, which was below the levels prescribed by EU regulations. This data demonstrated a high sensitivity of a singleplex EPFS PCR assay for detecting GM maize.

#### Construction of Standard Curves and Quantitative Detection

Five concentrations from 10^3^ to 10^7^ copies of four GM maize and endogenous gene were used to construct singleplex standard curves with NTC as negative control (see Fig. S2 for singleplex standard curves). Five standard curves showed a typical log-linear standard curve between the log copy numbers and the determined RFU values. The regression correlation coefficient (R^2^) values were >0.9982, which indicated excellent relationship. The absolute LOD of singleplex EPFS method was 10^3^ copies for detection of five types of samples. All this suggested that the new method is highly accurate and it could be used to quantify the event-specific gene of BT176 maize (Fig. S2a), GA21 maize (Fig. S2b), NK603 maize (Fig. S2c), TC1507 maize (Fig. S2d) and the IVR gene of maize (Fig. S2e).

### BT176, GA21, NK603 and TC1507 Multiplex Assay

#### Specificity Assay, Qualitative Detection and High Throughput

**Figure 3:**
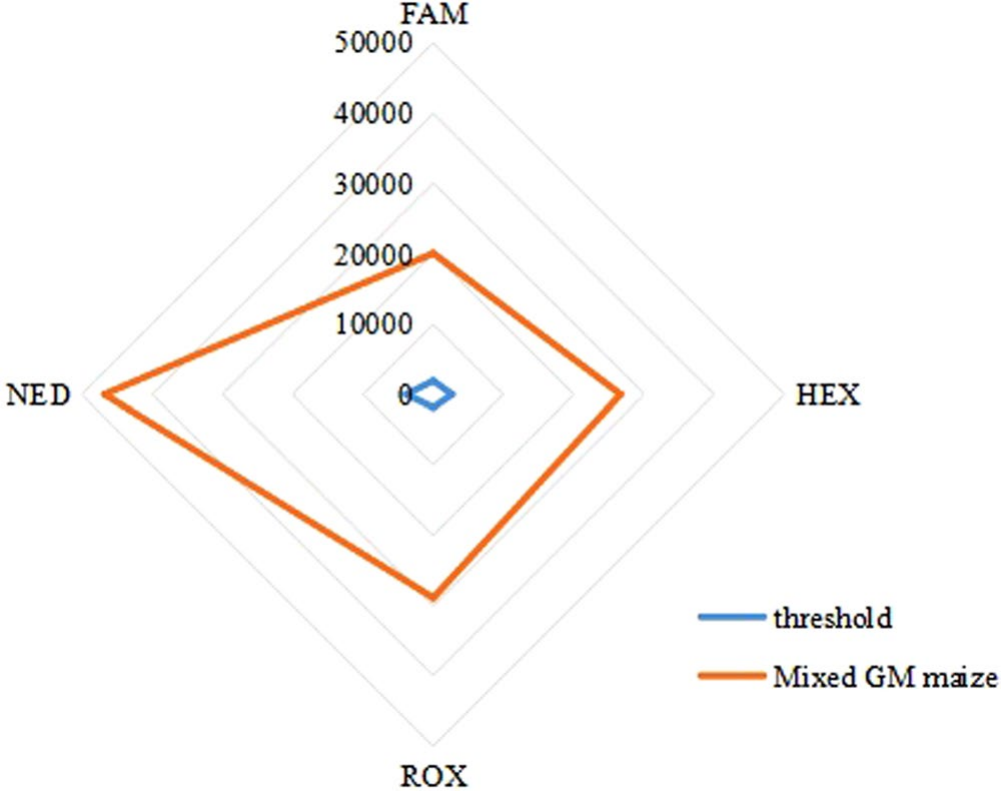
Multiplex specificity assay for detection of four GM maize simultaneously using EPFS method in a single reaction. Four pairs of fluorescent-labeled primers were used to amplify four event-specific genes of four GM maize. Four fluorescent-labeled amplicons were analyzed to evaluate the multiplex specificity of the EPFS method. FAM: labeled BT176 amplicons; HEX: labeled GA21 amplicons; ROX: labeled NK603 amplicons; NED: labeled TC1507 amplicons; RFU: relative fluorescence units.

Specificity of the 4-plex EPFS were confirmed on the mixed DNA materials containing four GM events (BT176 maize, GA21 maize, NK603 maize and TC1507 maize) in a single reaction. Results are shown in Fig. 3. The amplification results of four maize genomes showed positive signals demonstrating high specificity of 4-plex EPFS with high throughput. Overall, these data showed that increase in the number of target DNAs to be analyzed in the EPFS method did not affect the results of qualitative detection.

#### Sensitivity Assay

**Figure 4:**
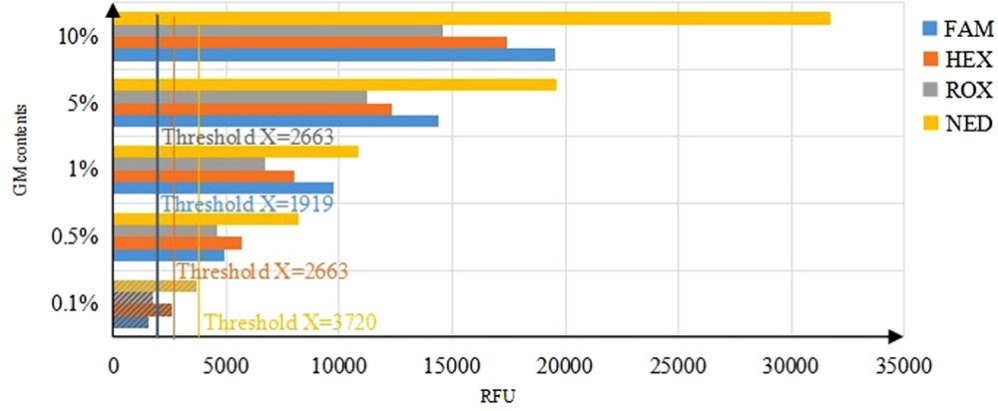
Sensitivity of the multiplex EPFS assay for GM maize detection. Multiplex EPFS assay were performed on a series of DNA solution with a final relative content of 10%, 5%, 1%, 0.5% and 0.1% (w/w). The column with slash means negative, which was lower than the threshold. FAM: labeled BT176 amplicons; HEX: labeled GA21 amplicons; ROX: labeled NK603 amplicons; NED: labeled TC1507 amplicons; RFU: relative fluorescence units.

The sensitivity of 4-plex EPFS method was further tested using mixed genomic DNAs of four GM maize at different levels (fortified at 10%, 5%, 1%, 0.5% and 0.1% (w/w)), respectively (Fig. 4). All four fluorescent-labeled amplicons could be simultaneously detected and showed positive signals, except for 0.1% GMO content. The results suggested that four GM maize could be detected simultaneously starting from 0.5% level (relative LOD), which was also lower than levels prescribed by EU regulation and was consistent with singleplex analysis results. To sum up, this data indicated that the 4-plex EPFS method could be used to detect GM content with high sensitivity and high throughput.

#### Construction of Standard Curves and Quantitative Detection

**Figure 5:**
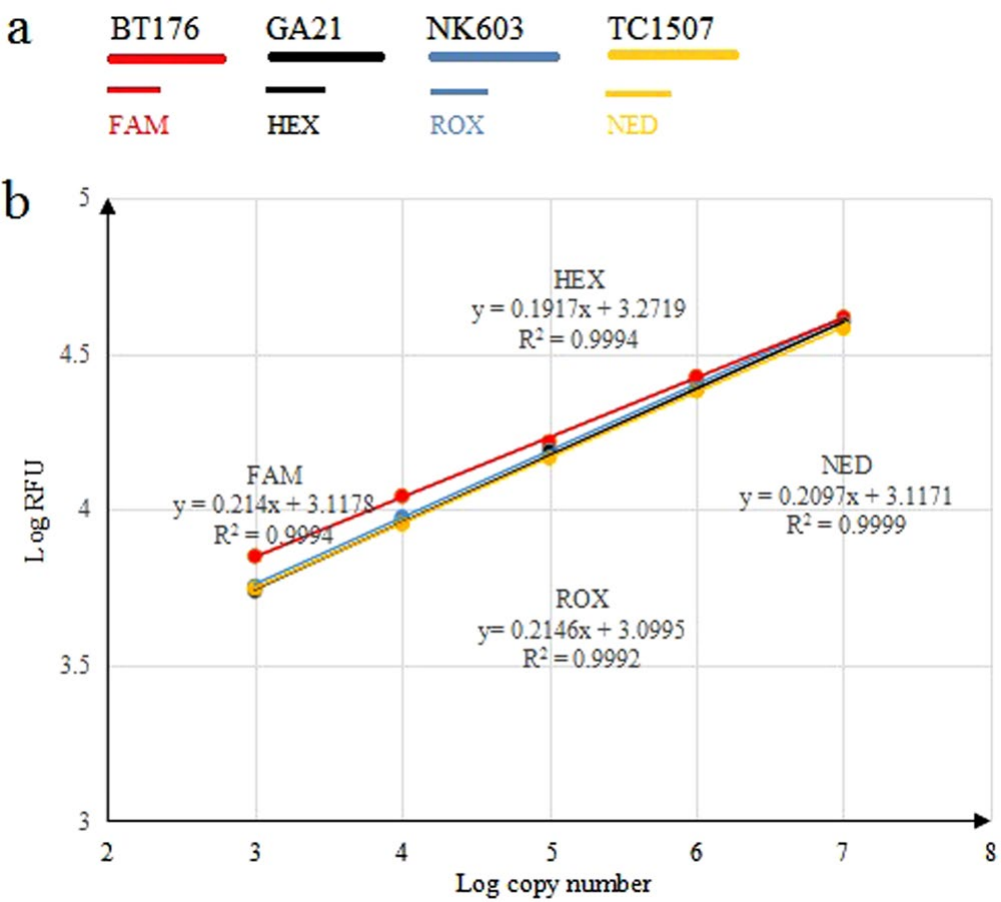
The EPFS assay was used to quantitatively analyze the GM maize. (**a**) Four sets of labeled primers targeted amplicons at event-specific gene of four different GM maize. (**b**) When run in a multiplex reaction, it is obvious that the amplification efficiencies of the four reactions are approximately the same, with similar x-intercepts indicating roughly similar sensitivity. RFU: Relative fluorescence units.

Standard curves of 4-plex EPFS assay were established on a series of serial diluted DNA solutions (10^7^–10^3^ copies) containing event-specific gene of four GM maize (Fig. 5). The typical linear standard curves of four GM maize showed an excellent linearity between the logarithm of RFU and copy numbers. The absolute LOD of the 4-plex EPFS method is 10^3^ copies. These results are consistent with singleplex EPFS assay, indicating that 4-plex EPFS PCR assay has high accuracy and could be used to quantify the GM maize with high throughput.

### Repeatability

The repeatability of the 4-plex EPFS system for detection of GM maize was measured by ten intra-assays and ten inter-assays at 10^6^ copies for each of GM maize (see Table S1 for repeatability). As a result, the coefficient of variation (CV) was between 0.6–2.3% in the same batch, and between 9.6–14.5% in the ten different batches, thus fully meeting the acceptance criteria.

### The Detection of Simulated Samples

To validate the accuracy and precision of EPFS method, four simulated samples were tested. Each sample was repeatedly amplified in triplex. After that, the mean RFU were determined and used to calculate the corresponding copies through the formula from the corresponding standard curve. The simulated percentage was calculated according to the ratio of the copy numbers of GM maize to the copy numbers of IVR gene. As a result, the calculated GM contents from experiments showed a slight deviation from the known GM contents (Table S2 for simulated samples). Thus, this method was accurate and precise enough to evaluate the GM contents in practical samples.

### Reproducibility

To evaluate the applicability of EPFS method, other four foodborne pathogenic bacteria were also tested. This 4-plex EPFS assay for detection of foodborne pathogenic bacteria was performed and analyzed in the manner of EPFS system described above, except for the PCR amplification system.

#### Specificity Assay, Qualitative Detection and High Throughput

The DNA mixtures containing four foodborne pathogenic bacteria were used as template to evaluate the specificity of the 4-plex EPFS. The results showed that the RFU values for testing of four foodborne pathogenic bacteria were higher than the corresponding threshold (Fig. S3 for specificity assay). Therefore, the 4-plex EPFS assay for detection of four foodborne pathogenic bacteria also showed high specificity. This indicated that the EPFS method could also be applied to qualitatively detect multiple genes in other biological samples.

#### Construction of Standard Curves and Quantitative Detection

Standard curves of 4-plex EPFS assay for detection of four foodborne pathogenic bacteria were established on serial diluted plasmids. The logarithm of RFU values and the target gene copy number showed excellent linear relationship for detection of four foodborne pathogenic bacteria (Fig. S4 for standard curves). The absolute LOD was determined to be 10^3^ copies. Again, the results were consistent with the results of 4-plex EPFS assay for detection of four GM maize samples, implying its potential use for quantifying other biological genes with high throughput.

#### Repeatability

The repeatability of the EPFS system for testing the four foodborne pathogenic bacteria were studied by ten intra-assays and ten inter-assays at 10^6^ copies of targeted genes. The coefficient of variation (CV) was between 1.2–2.2% in the same batch and between 9.1–11.8% among the ten different batches, which was in line with the repeatability assay for testing the four GM maize (see Table Supp 3 for repeatability).

Altogether, the obtained results demonstrated good specificity, sensitivity, linearity and repeatability of the EPFS method which was developed in our laboratory, and which could be used for qualitative, quantitative and high-throughput analysis of multiple genes in a single reaction.

## Discussion

In this study, we established and validated a novel emulsion PCR method combined with fluorescence spectrophotometry for simultaneous qualitative, quantitative and high-throughput detection of multiple DNA targets. The sensitivity and specificity of the system were examined using four kinds of GM maize. By labeling different gene-specific primer pairs with different fluorophores that did not interfere with each other, multiple target DNAs could be co-amplified in a single PCR reaction, and amplicons could be qualitatively and quantitatively analyzed with high throughput. The EPFS method showed high sensitivity (0.5% (w/w)) which was below the standard levels proposed by EU criteria. In addition, excellent linearity relationship between the logarithm of RFU values and gene copy numbers were detected, and the absolute LOD of EPFS was 10^3^ copies. The 4-plex method increased the throughput via emulsion PCR reaction but without compromising the quality of resulting data. The slight deviation between the calculated GM contents and simulated GM contents confirmed the precision and accuracy of this EPFS method. Furthermore, the qualitative, quantitative and high-throughput detection results obtained from four foodborne pathogenic bacteria further confirmed the applicability of EPFS method to other biological samples.

The excellence of EPFS method can be attributed to the combination of emulsion PCR and fluorescence spectrophotometry. Emulsion PCR can improve the throughput, thus avoiding constraints of multiple PCRs by partitioning the reaction mixture into discrete droplets while maintaining the specificity and sensitivity. Emulsion PCR was first systematically described by Williams *et al*. who have amplified complex DNA mixtures by compartmentalization of genes in water-in oil (w/o) emulsion^19^. Emulsion PCR method consists of emulsion PCR and analysis of the products. Williams and his team have used agarose gel electrophoresis to analyze the PCR products; nonetheless, their method could only be used for qualitative analysis but not for quantitative determination^19^. In addition, the contamination derived from the agarose gel electrophoresis appeared to be defective. Later on, Guo *et al*. have used microdreoplet PCR implemented capillary gel electrophoresis to amplify and analyze multiple DNA targets^4^; however, their two-step PCR method containing preamplification PCR and emulsion PCR has shown to be relatively complicated and not suitable for quantitative determination. Droplet digital PCR enables absolute quantitation of low copy number nucleic acid samples with an automated droplet generator; though, the low throughput and high cost hamper its widespread use^20^. In the present study, we combined the emulsion PCR with fluorescence spectrophotometry to amplify and analyze multiple targeted DNA qualitatively and quantitatively with high throughput at reasonable cost. The emulsion PCR products were directly measured by Infinite M1000 PRO to avoid the contamination derived from the agarose gel electrophoresis. Conventional PCR could only be used for qualitative detection, but not for quantitative detection, and the throughput was limited^21^. While real-time quantitative PCR enables quantitative detection with low LOD and high accuracy, its throughput is incomplete due to limited detecting channels in real-time systerm^22^. The EPFS method used Infinite M1000 PRO to measure the RFU of the fluorophore, which can detect the emission wavelength from 200 nm to 1000 nm (Thermo VarioSkan Flash), i.e. 12 fluorophores can be analyzed in a well. With appropriate adjustment, almost 20 fluoropores could be tested simultaneously in a well, which means more than 6000 genes could be detected in a 384-well plate at a time. Therefore, our EPFS method could detect abundant target genes simultaneously.

DNA microarray is suitable for high-throughput and qualitative analysis, but not for quantitative assay due to the poor linearity. In addition, preparation of high-density array is time consuming and the confocal laser scanner is very expensive^17^. In the process of amplification using microarray, it was easy to contaminate the target samples thus affecting signal-to-noise^17^. By contrast, EPFS method only requires conventional thermocycle instrument and a fluoro-microplate reader (or fluorospectrophotometer) to qualitatively and quantitatively detect multiple target DNAs with high throughput, clearly indicating its simplicity, speediness, concision and low cost.

Many emulsion PCR techniques have currently adopted a known instrumentation and technologies for DNA analysis, such as agarose gel electrophoresis^19^, microarray^7^, capillary electrophoresis^4^, and micro-fluidic chip^23^. To the best of our knowledge, the new EPFS system is the first analytical technology of this kind that enables simultaneous qualitative, quantitative and high-throughput analysis of multiple genes. The excellent performance of the EPFS method enables its application in the field of animal husbandry, including the detection of veterinary pathogenic microorganisms, human pathogenic microorganisms, aquatic pathogenic microorganisms, environmental and plant pathogenic microbes. The EPFS method could also be used for detection of varieties of human disease-related genes and genetic counseling. The excellent performance of EPFS system described in this study is still dimmed by the manual purification of emulsion products. This, however, can be improved with the use of automatic purification system, by which time the repeatability and accuracy should be greatly improved.

## Supplementary information

Supplementary Information
