# Supplementary material for: A novel emulsion PCR method coupled with fluorescence spectrophotometry for simultaneous qualitative, quantitative and high-throughput detection of multiple DNA targets
Source: Sci Rep. 2019 Jan 17;9:184. doi: 10.1038/s41598-018-36981-1 (PMC6336815; doi:10.1038/s41598-018-36981-1)
Supplement: Supplementary file 1 — Supplementary Information [file 41598_2018_36981_MOESM1_ESM.pdf]

A novel emulsion PCR approach coupled with fluorescence spectrophotometry for simultaneous qualitative, quantitative and high-throughput detection of multiple DNA targets

Yanan Du[3 +] , Xiao Zhao[1,2 +] , Binan Zhao[4], Yan Xu[3],Wei Shi[3], Fangfang Ren[3], Yangyang Wu[3], Ruili Hu[3], Xiaorui Fan[3], Qi Zhang[3], Xiaoxia Zhang[3],Wanjing Zhang[3], Wenjing Wu[3], Bin Shi[5], Huanzhen Zhao [5]&Kai Zhao[1, 2 *]

[1]Biotechnology Research Institute, Shanghai Academy of Agricultural Sciences, 2901 Beidi Road, Shanghai 201106, China. [2]Key Laboratory of Agricultural Genetics and Breeding, Shanghai Academy of Agricultural Sciences, 2901 Beidi Road, Shanghai 201106, China. [3]College of Life and Environment Sciences, Shanghai Normal University, 100 Guilin Road 200234, Shanghai, China. [4]Department of Clinical Medicine, XuZhou Medical University, 209 Tongshan Road 221004, Xuzhou, China. [5]Shanghai Bio-full Biotech Co.,Ltd, 2901 Beidi Road, Shanghai 201106, China.

[+]Y.N.D. and X.Z. contributed equally to this work.
[*]Correspondence should be addressed to K.Z. (kzhao118@163.com)

Supplementary information

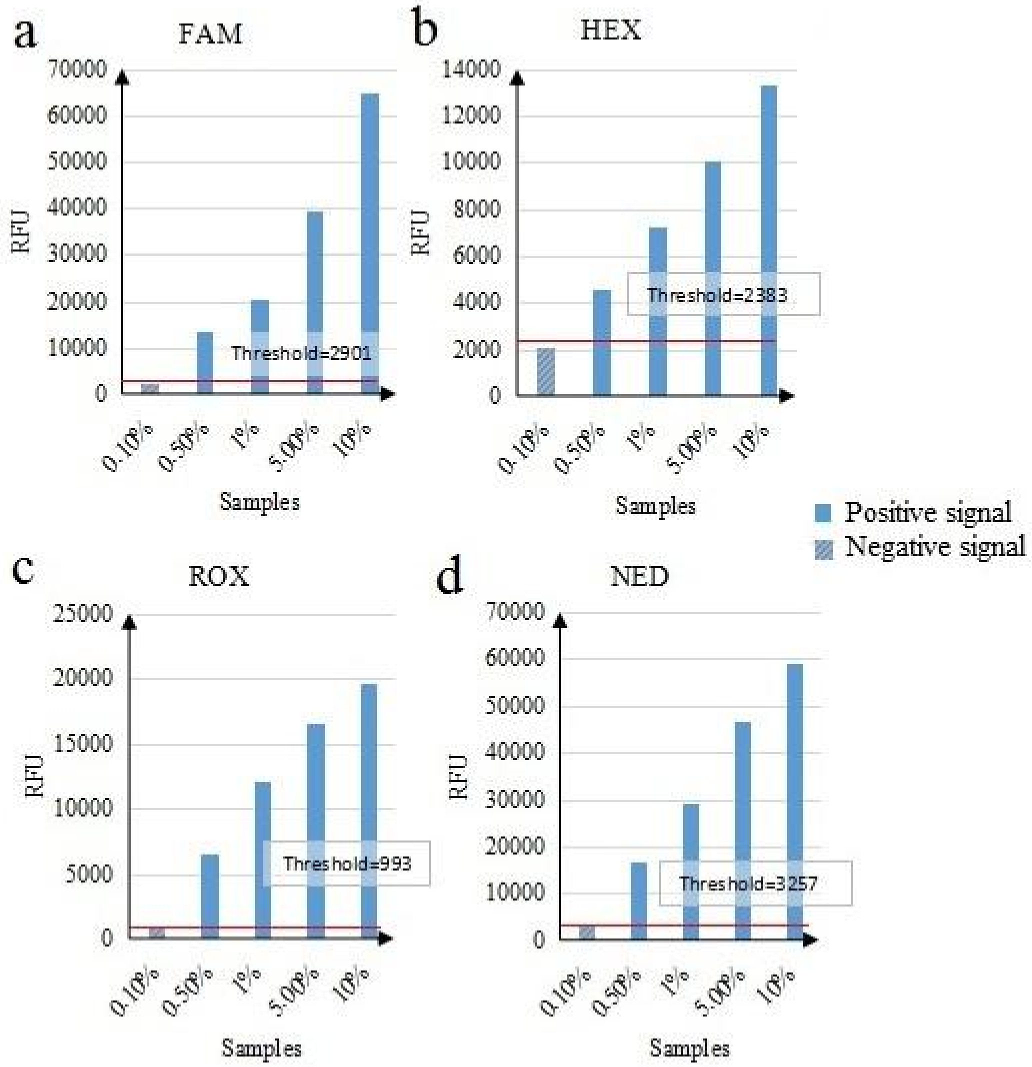

Figure Supp1. Sensitivity of the singleplex EPFS assay for GM maize detection. (a) The singleplex sensitivity assay for detection of BT176 GM maize. The forward primer of BT176 were labeled with FAM to amplify DNA samples with different GM content to determine the relative limit of detection of BT176 by EPFS method. **(b)** The singleplex sensitivity assay for detection of GA21 GM maize. The forward primer of GA21 were labeled with HEX to amplify DNA samples with different GM content to determine the relative limit of detection of GA21 by EPFS method. **(c)** The singleplex sensitivity assay for detection of NK603 GM maize. The forward primer of NK603 were labeled with ROX to amplify DNA samples with different GM content to determine the relative limit of detection of NK603 by EPFS method. **(d)** The singleplex sensitivity assay for detection of TC1507 GM maize. The forward primer of TC1507 were labeled with NED to amplify DNA samples with different GM

content to determine the relative limit of detection of TC1507 by EPFS method. RFU: relative fluorescence units.

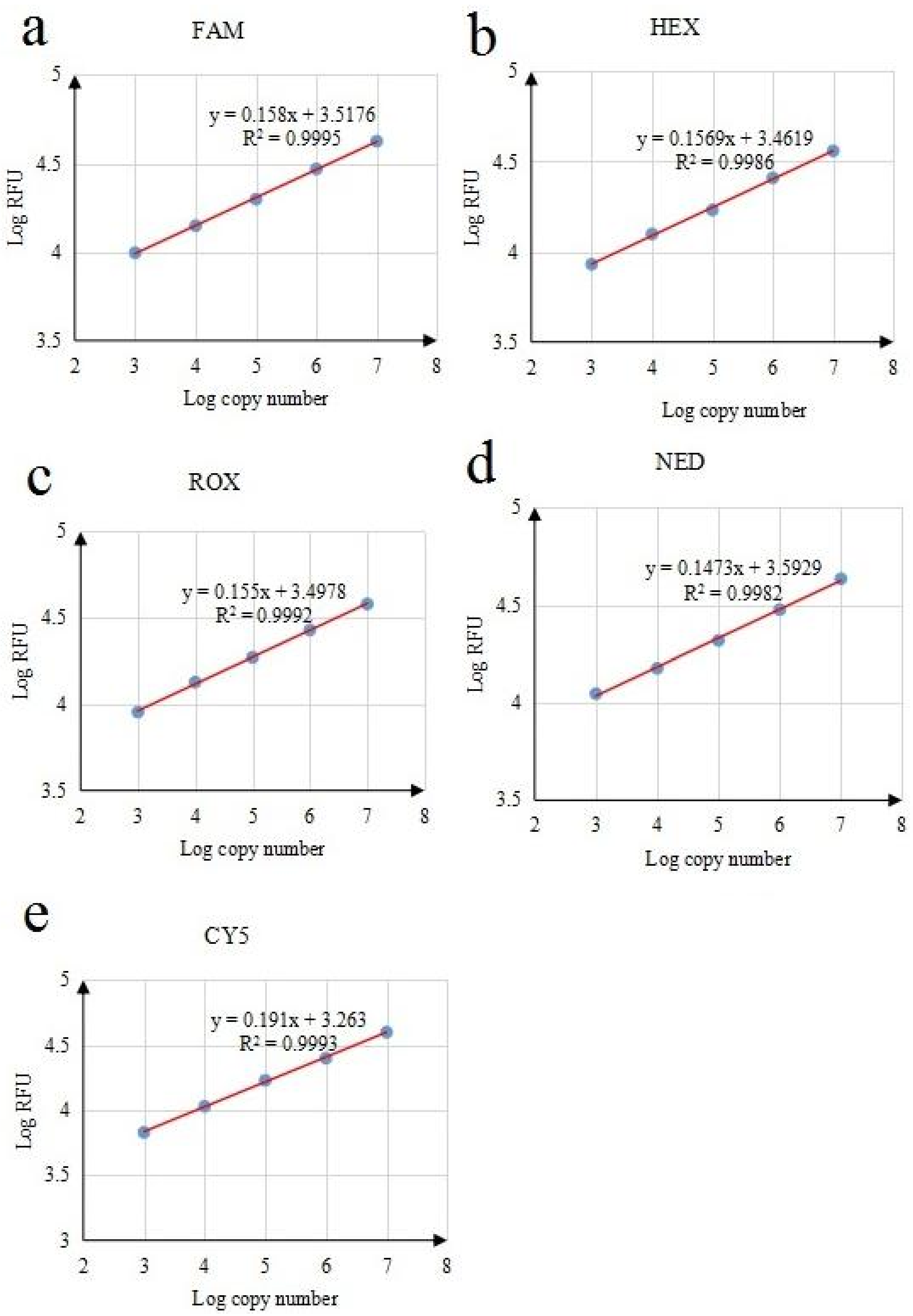

Figure Supp2. Singleplex standard curves for four GM maize and IVR gene of

maize. **(a)** The forward primer of BT176 were labeled with FAM to construct standard curve on a series of serial diluted plasmids for quantitative analysis of samples contained BT176 content. **(b)** The forward primer of GA21 were labeled with HEX to construct standard curve on a series of serial diluted plasmids for quantitative analysis of samples contained GA21 content. **(c)** The forward primer of NK603 were labeled with ROX to construct standard curve on a series of serial diluted plasmids for quantitative analysis of samples contained NK603 content. **(d)** The forward primer of TC1507 were labeled with NED to construct standard curve on a series of serial diluted plasmids for quantitative analysis of samples contained TC1507 content. RFU: Relative fluorescence units.

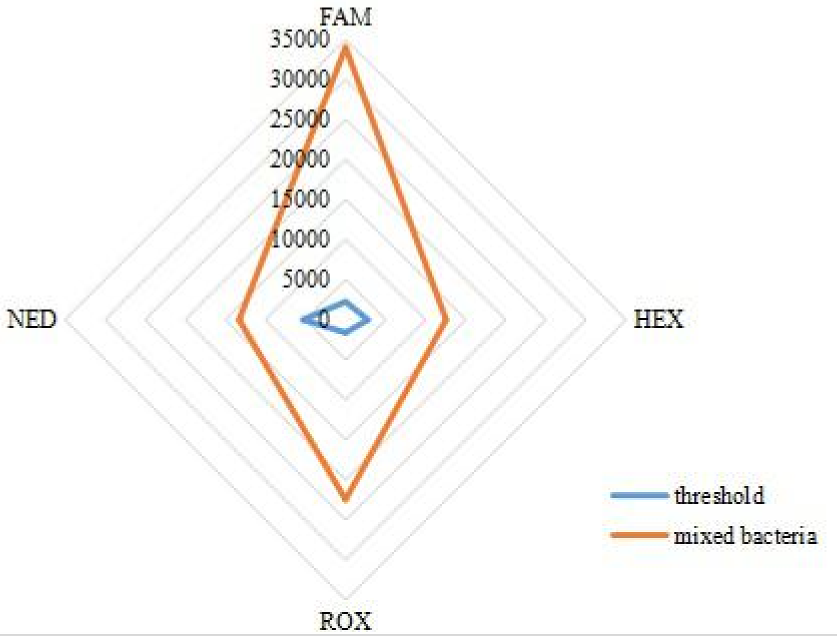

Figure Supp3. Multiplex specificity assay for detection of four foodborne pathogenic bacteria simultaneously using EPFS method in a single reaction. Four pairs of fluorescent-labeled primers were used to test each of the foodborne pathogenic bacteria. Four fluorescent-labeled amplicons were analyzed to evaluate the multiplex specificity of the EPFS method. FAM: labeled Salmonella amplicons; HEX: labeled Listeria monocytogenes amplicons; ROX: labeled Escherichia coli amplicons; NED: labeled Staphylococcus aureus; RFU: relative fluorescence units.

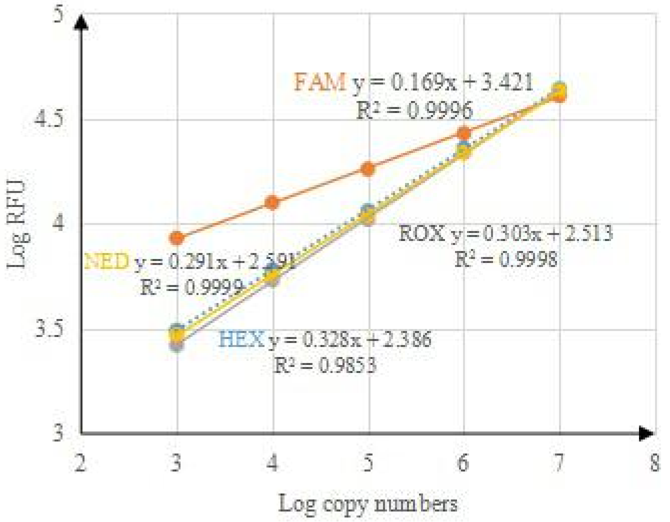

Figure Supp4. Four foodborne pathogenic bacteria multiplex standard curves. FAM: labeled Salmonella amplicons; HEX: labeled Listeria monocytogenes amplicons; ROX: labeled Escherichia coli amplicons; NED: labeled Staphylococcus aureus; RFU: relative fluorescence units.

Table Supp1. Repeatability of EPFS method for detection of four GM Maize

Samples	Intra-assay(RFU)			Inter-assay(RFU)		
	AVE	SD	CV%	AVE	SD	CV%
BT176	39855.2	932.26	2.34	38027.7	3641.87	9.58
GA21	38587.7	658.87	1.71	37467.2	4145.06	11.06
NK603	38846.3	469.82	1.21	36877.9	145.06	11.24
TC1507	38846.3	219.25	0.62	34649.3	5036.17	14.53

AVE: Mean relative fluorescence units; SD: Standard deviation; CV: Coefficient of variation; RFU: Relative fluorescence units.

Table Supp2. Quantification of four GM maize content in three simulated samples

Samples	Fluorophores	RFU			AVE	SD	Copies	GM%
		1	2	3				
BT176 MPFS assay								
T1 (1%)	FAM	16490	15282	15697	15823.00	501.15	113158.22	1.06
T2 (3%)	FAM	18842	19676	18961	19159.67	368.32	276694.01	2.95
T3 (5%)	FAM	22453	21604	21916	21991.00	350.64	526872.47	5.03
GA21 MPFS assay								
T1 (1%)	HEX	16929	17966	17073	17322.67	458.69	110370.27	1.03
T2 (3%)	HEX	21560	19942	20470	20657.33	673.70	276504.32	2.95
T3 (5%)	HEX	22972	23029	23947	23316.00	446.79	519976.30	4.96
NK603 MPFS assay								
T1 (1%)	ROX	14721	15764	14816	15100.33	470.88	107207.75	1.00
T2 (3%)	ROX	19064	18180	18214	18486.00	408.94	275183.45	2.93
T3 (5%)	ROX	20906	22019	20842	21255.67	540.39	527419.58	5.03
TC1507 MPFS assay								
T1 (1%)	NED	15283	14942	14579	14934.67	287.45	109897.97	1.03
T2 (3%)	NED	17904	18415	18302	18207.00	219.16	282685.63	3.01
T3 (5%)	NED	20930	20956	20302	20729.33	302.36	524815.15	5.01
IVR MPFS assay								
T1	CY5	39570	40607	40288	40155	433.67	10692504.1	
T2	CY5	41284	36254	39960	39166	2128.86	9380519.15	
T3	CY5	40822	40539	38636	39999	970.69	10476239.7	

T1: 1% BT176, 1% GA21, 1% NK603, 1% TC1507, 96% Non-GM maize. T2: 3% BT176, 3% GA21, 3% NK603, 3% TC1507, 88% Non-GM maize. T3: 5% BT176, 5% GA21, 5% NK603, 5% TC1507, 80% Non-GM maize ; RFU: Relative fluorescence units; AVE: Mean relative fluorescence units; SD: standard deviation; CV: coefficient of variation; GM% : Genetically modified maize content.

Table Supp3. Repeatability of EPFS method for detection of of foodborne pathogenic bacteria

10^6 Copies Plasmids	Intra-assay			Inter-assay		
	AVE	SD	CV%	AVE	SD	CV%
Salmonella	36065	963.43	2.67	36159.8	4253.83	11.76
L. monocytogenes	39208.3	579.29	1.48	37504.1	3400.74	9.07
E. coli	38827.9	473.30	1.22	39647.6	4231.33	10.67
S. aureus	38952.9	596.13	1.53	35523.1	4147.47	11.68

AVE: mean relative fluorescence units; SD: standard deviation; CV: coefficient of variation; L. monocytogenes: Listeria monocytogenes; E. coli: Escherichia coli; S. aureus: Staphylococcus aureus.
